# Antimicrobial resistance characteristics and associated molecular mechanisms of clinically isolated *Haemophilus influenzae* from the lower respiratory tract in Chongqing, China

**DOI:** 10.3389/fcimb.2026.1785829

**Published:** 2026-06-03

**Authors:** Qiong Huang, Rong Xiang, Yong Wu, Wenfeng Zhang, Wei Xie, Zhiyong Liu, Nian Quan, Yuan Gao, Zhongzheng Xiong, Li He, Jing Fu, Di Mu, Yunying Wang

**Affiliations:** 1 Department of Laboratory Medicine, The Second Affiliated Hospital of Chongqing Medical University, Chongqing, China; 2 Precision Medicine Center, The Second Affiliated Hospital of Chongqing Medical University, Chongqing, China; 3 Department of Laboratory Medicine, Fengdu General Hospital, Chongqing, China; 4 Department of Hepatobiliary Surgery, The Second Affiliated Hospital of Chongqing Medical University, Chongqing, China; 5 Department of Laboratory Medicine, Children’s Hospital of Chongqing Medical University, Chongqing, China; 6 Department of Laboratory Medicine, The First Affiliated Hospital of Army Medical University, Chongqing, China; 7 Department of Laboratory Medicine, The Second Affiliated Hospital of Army Medical University, Chongqing, China; 8 Department of Laboratory Medicine, Chongqing University Jiangjin Hospital, School of Medicine, Chongqing University, Chongqing, China; 9 Department of Laboratory Medicine, Dianjiang People’s Hospital of Chongqing, Chongqing, China; 10 Department of Laboratory Medicine, People’s Hospital of Shizhu Tujia Autonomous County, Chongqing, China; 11 Department of Laboratory Medicine, Zhongxian People’s Hospital of Chongqing, Chongqing, China

**Keywords:** *Haemophilus influenzae*, BLNAR, antibiotic resistance, multi-center surveillance, resistance mechanisms, whole-genome sequencing

## Abstract

**Background:**

Global systematic research on *Haemophilus influenzae* drug resistance profiles and mechanisms is scarce. Many studies are constrained by single-center designs and restricted sample numbers, hindering the proper monitoring of drug resistance trends and the analysis of drug resistance mec e antimicrobial resistance mechanisms.

**Results:**

Ampicillin had the highest resistance rate (76.2%), followed by azithromycin (38.2%) and amoxicillin/clavulanic acid (23.8%); tetracycline (6.2%) and levofloxacin (0.6%) had low resistance rates. No resistance to ceftazidime, cefotaxime, or meropenem was detected. The β-lactamase positive rate was 67.2%. The *TEM* gene detection rate was 67.2%, frequently coexisting with *mef(A)*, *msr(D)*, and *tet(B)* to form the local core resistome. Resistance mechanisms: ampicillin resistance (β-lactamase + *ftsI*/PBP3 mutations); macrolide resistance (*mef(A)*/*msr(D)* efflux pumps); tetracycline resistance (*tet(B)*); levofloxacin resistance (multiple *ParC* amino acid substitutions).

**Conclusion:**

Chongqing *H. influenzae* isolates had high ampicillin resistance, while the resistance to azithromycin and amoxicillin/clavulanate cannot be ignored. The underlying resistance mechanisms were marked by the co-occurrence of macrolide and tetracycline-resistance genes on a *TEM* gene background. Continuous local surveillance is warranted to optimize empirical antimicrobial therapy and anticipate emerging resistance risks.

## 1 Introduction

*Haemophilus influenzae* (*H. influenzae*, Hi) is a small, Gram-negative coccobacillus that commonly colonizes the upper respiratory tract. Infants and young children(especially those under five years)are the primary hosts due to their immature immune systems, which confer heightened susceptibility (Zhu et al., 2015). *H. influenzae* is a leading cause of community-acquired pneumonia in children (Zhu et al., 2015; Tran et al., 2024). Transmission occurs through close contact and respiratory secretions. The organism is frequently isolated from clinical specimens such as sputum, blood, and cerebrospinal fluid. *H. influenzae* infection is localized and seasonal, with peak prevalence during winter and spring (Ma et al., 2023). It can lead to respiratory tract infections (for example, pneumonia and otitis media), invasive disease (for example, septicemia and meningitis), infections of the urinary and reproductive systems, and circulatory system infections. Before the introduction of vaccines, the significant capsular toxicity of *H. influenzae* type b (Hib) was the primary etiological factor for invasive infections. The global vaccination and promotion of the Hib conjugate vaccine have significantly reduced the nasopharyngeal colonization rate of Hib and the incidence of associated infections, whereas non-typeable *H. influenzae* (NTHi) has emerged as a significant pathogen in bacterial pneumonia. Thus, the World Health Organization categorizes *H. influenzae* as a pathogen of medium priority (Sati et al., 2025).

Nonetheless, systematic studies of *H. influenzae* antimicrobial resistance mechanisms in China remain limited. Previous epidemiological investigations in Beijing (Zhu et al., 2015), Shanghai (Li et al., 2020), Guangzhou (Wen et al., 2023), Guiyang (Zhou et al., 2022), and Kunming (Yuan et al., 2023) have been constrained by single-center designs, small sample sizes (typically <100 isolates), and reliance on phenotypic susceptibility testing with limited genotyping. International genomic studies have advanced our understanding of *H. influenzae ftsI*-mediated beta-lactam resistance (Takahata et al., 2007), yet these investigations were conducted in European and Japanese populations and may not reflect the distinct epidemiological characteristics and resistance profiles of Chinese clinical isolates. To address these gaps, we conducted a multi-center study of 500 *H. influenzae* isolates from 9 hospitals in Chongqing, integrating whole-genome sequencing with comprehensive phenotypic profiling. Our study advances beyond previous work in three key respects: (1) Scale — this represents the largest WGS-based *H. influenzae* resistance study in southwestern China, with 500 isolates from 9 hospitals spanning urban and rural areas; (2) Methodology — we simultaneously characterize the resistome, mobilome, and population structure using a unified genomic framework, rather than relying on targeted genotyping of individual resistance determinants; and (3) Biological insight — we perform multi-scale phylogenomic analyses (local n=500, national n=589, and global n=5, 594) to contextualize Chongqing isolates within the broader Chinese and global *H. influenzae* population, revealing novel co-resistance modules (*blaTEM*–*mef(A)*/*msr(D)*–*tet(B)*) and *ftsI* allelic diversity (11 distinct alleles) not previously described in Chinese clinical settings.

## 2 Materials and methods

### 2.1 Isolate collection

Building on the “Chongqing Specialty Alliance for Laboratory Diagnosis of Pathogenic Microorganisms, ” A total of 500 bacterial strains were collected from patients across various regions, hospital stages, and populations at 9 representative medical centers in Chongqing from January 2024 to April 2025. The distribution of strains is as follows: The Second Affiliated Hospital of Chongqing Medical University (n=121), Children’s Hospital of Chongqing Medical University (n=77), The First Affiliated Hospital of Army Medical University (n=33), The Second Affiliated Hospital of Army Medical University (n=28), Chongqing University Jiangjin Hospital (n=55), Fengdu General Hospital (n=136), Dianjiang People’s Hospital of Chongqing (n=28), People’s Hospital of Shizhu Tujia Autonomous County (n=11), and Zhongxian People’s Hospital of Chongqing (n=11). Duplicate specimens from the identical patient, originating from the same site or source, were eliminated. All strains were identified by the matrix-assisted laser desorption ionization time-of-flight mass spectrometry (MALDI-TOF MS) automatic bacterial identification system (Bruker Daltonics, Bremen, Germany). After collection, the strains were stored in a -80°C refrigerator and revived prior to testing.

### 2.2 β-Lactamase detection

After reviving the bacterial strain, smear the colonies of *H. influenzae* onto cefinase test discs and observe the results after 10 minutes. A color change of the disc from yellow to red is interpreted as a β-lactamase-positive result; no color change indicates a negative result.

### 2.3 Broth microdilution susceptibility testing

The antibiotics ampicillin, amoxicillin/clavulanate, ceftazidime, cefotaxime, meropenem, levofloxacin, azithromycin, and tetracycline were used to test the sensitivity of drugs to bacterial strains. Antimicrobial susceptibility testing were performed using the broth microdilution method according to CLSI M07 (CLSI, 2024). MIC results were interpreted using CLSI M100 (35th edition, 2025) (CLSl, 2025). The test medium was cation-adjusted Mueller–Hinton broth (Becton, Dickinson and Company, BD) supplemented with yeast extract (Oxoid), β-NADH (V factor; Sigma-Aldrich), and hemin (X factor; TCI). Antimicrobial agents were obtained from the National Institutes for Food and Drug Control (NIFDC, China). *H. influenzae* ATCC 49247 was used for quality control.

### 2.4 Whole Genome Sequencing

#### 2.4.1 WGS and genome assembly

Genomic DNA was extracted from single-colony *H. influenzae* isolates obtained from 500 patients using the TIANamp^®^ Bacteria DNA Kit, according to the manufacturer’s protocol. DNA purity was assessed with a NanoDrop™ spectrophotometer (A260/280 ratio 1.8–2.0), and DNA concentration was quantified using Qubit. Qualified DNA was submitted to Suzhou Hongxun Biotechnology Co., Ltd. (Suzhou, China) for library construction and sequencing. Only samples meeting the pre-defined criteria (total DNA >2 μg and predominantly high-molecular-weight fragments >20 kb) were advanced to library preparation. DNA was fragmented by Covaris ultrasonication with a target insert size of 350–450 bp. Libraries were prepared using the NEBNext^®^ Ultra™ II DNA Library Prep Kit (Illumina^®^). Dual-sided size selection and purification were performed using AMPure XP beads to constrain the fragment-size window. Libraries were quantified by qPCR and fragment-size distributions were verified with an Agilent 2100 Bioanalyzer (main peak 500–600 bp including adapters). Paired-end sequencing (2 × 150 bp) was conducted on an Illumina NovaSeq 6000 platform. The desired coverage depth was ≥100× per isolate, with around 1% PhiX included as an internal control.

Raw reads were initially assessed for quality using FastQC (v0.11.9)^1^, followed by adapter trimming and quality filtering with fastp (v0.20.1) (Chen et al., 2018). *De novo* genome assembly was performed using SPAdes (v3.15.5) (Bankevich et al., 2012), and assembly quality metrics were evaluated using QUAST (Gurevich et al., 2013).

#### 2.4.2 Collection and integration of *H. influenzae* genomic data

To support phylogenetic and comparative genomic analyses, we assembled three genomic datasets. The local dataset comprised 500 *H. influenzae* genomes newly generated in this study. The China dataset included the local genomes together with 89 publicly available *H. influenzae* genomes from mainland China retrieved from NCBI GenBank (downloaded as of September 2025). The global comparative dataset integrated the local (n=500) and Chinese public genomes (n=89) with an additional 5, 005 complete *H. influenzae* genomes sampled worldwide, yielding a total of 5, 594 genomes for global comparison.

#### 2.4.3 Annotation, feature analysis, and phylogenetic reconstruction of genomic datasets

For all assemblies, contigs shorter than 1, 000 bp or with mean coverage depth <5× were removed prior to downstream analyses. Genome quality was assessed using CheckM (v1.2.2) (Parks et al., 2015), and only genomes meeting the predefined thresholds (completeness >95% and contamination <5%) were retained. Genome annotation, both structural and functional, was conducted utilizing Prokka (v1.14.6) (Seemann, 2014).

Acquired antimicrobial resistance genes were identified using ResFinder (v4.1) (Bortolaia et al., 2020) with minimum thresholds of sequence identity >90% and coverage >80%, which are established community standards validated across diverse bacterial species (Bortolaia et al., 2020). These thresholds are more stringent than the current ResFinder 4.0 defaults (80% identity/60% coverage) and balance sensitivity with specificity for acquired gene detection. For allele-level discrimination of specific resistance determinants (e.g., *ftsI* alleles encoding PBP3 variants), we applied more stringent criteria of ≥95% query coverage to ensure near-full-length sequence comparison, and required ≥1% nucleotide identity difference to define distinct alleles. The 1% identity difference threshold was empirically informed by the observed nucleotide divergence among functionally characterized PBP3 groups in *H. influenzae*: Group I, II, and III-like *ftsI* alleles differ by approximately 1–3% in the transpeptidase-encoding region (Ubukata et al., 2001; Dabernat et al., 2002; García-Cobos et al., 2007). This threshold avoids conflating functionally distinct alleles while appropriately collapsing variants differing only by synonymous substitutions. Mobile genetic elements were detected using MobileElementFinder (Johansson et al., 2021). Species identification was conducted using GTDB-Tk (v2.6.1) for taxonomic assignment (Chaumeil et al., 2019). Only the genomes that passed CheckM quality control and were unambiguously classified as *H. influenzae* by GTDB-Tk were included in subsequent analyses. Variant calling was performed using the *H. influenzae* reference genome FDAARGOS 1560 (GenBank accession CP085952.1). Reads were mapped and variants were called using Snippy (v4.6.0). Recombinant regions were identified and removed using Gubbins (v3.2.1) (Croucher et al., 2015), yielding a recombination-filtered core genome alignment, from which core Single Nucleotide Polymorphisms (SNPs) were extracted using SNP-sites (v2.5.1) (Page et al., 2016). A heatmap of resistance gene distribution was generated using the R package pheatmap. Maximum-likelihood phylogenies were inferred using IQ-TREE 2 under the GTR+F+I+G4 substitution model with 1, 000 ultrafast bootstrap replicates (Hoang et al., 2018; Minh et al., 2020), and trees were visualized and annotated using iTOL (v6.0) (Letunic and Bork, 2024).

#### 2.4.4 Bioinformatics pipeline and reproducibility

All bioinformatics analyses were performed using a standardized pipeline, with scripts and configuration files deposited in our GitHub repository. The key steps and parameters are summarized below and detailed in **Supplementary Table 3**. Quality control: Raw sequencing reads were quality-filtered using fastp (v0.23.4; parameters: --qualified_quality_phred 20 --length_required 50 --cut_front --cut_tail). *De novo* genome assembly was performed with SPAdes (v3.15.5; --careful mode). Assembly quality was assessed using QUAST (v5.2.0) and CheckM (v1.2.2), with completeness >95% and contamination <5% required for inclusion. Species confirmation: Species identity was confirmed using GTDB-Tk (v2.6.1) for taxonomic assignment against the Genome Taxonomy Database. Antimicrobial resistance gene detection: Acquired resistance genes were identified using ResFinder (v4.1; thresholds: sequence identity >90%, coverage >80%). Chromosomal point mutations associated with resistance were detected using PointFinder. *ftsI* gene alleles were extracted, aligned against the Rd KW20 reference (GenBank L42023.1), and classified according to the scheme of (Ubukata et al., 2001). Mobile genetic element analysis: Insertion sequences were identified using MobileElementFinder. Phylogenetic analysis: Core genome alignment was generated using Snippy (v4.6.0; --mincov 10 --minfrac 0.9) against reference genome FDAARGOS 1560. Recombination filtering was performed using Gubbins (v3.2.1). Maximum-likelihood phylogenetic trees were constructed using IQ-TREE 2 (v2.2.0; -m GTR+F+I+G4 -bb 1000). Trees were visualized and annotated using iTOL (v6). All custom scripts for data processing, statistical analysis, and figure generation are available in the GitHub repository with documentation.

### 2.5 Statistical analysis

A database was established to record each isolate’s antimicrobial susceptibility results and phenotypic characteristics and to calculate resistance rates (or non-susceptibility rates) for each antibiotic. The count data are expressed as counts and percentages. According to the study objectives, we conducted a stratified comparison of the resistance rates of bacterial strains from different demographic groups (children and adults) and regional hospitals (large tertiary hospitals in the main urban districts and county-level hospitals). Statistical analyses were performed in SPSS software version 26.0 (IBM). Categorical variables were analyzed using the chi-square (χ²) test or Fisher’s exact test. P-values below 0.05 were deemed statistically significant.

## 3 Results

### 3.1 Sample sources

A total of 500 *H. influenzae* isolates were collected from nine general hospitals in Chongqing during the years 2024–2025; all isolates were sourced from the lower respiratory tract. The patients are primarily from the pediatric department and the respiratory intensive care unit.

The sex distribution was 65.2% male (n=326) and 34.8% female (n=174), with a male/female ratio of 1.87 to 1, and the difference was statistically significant (p<0.05). Children (<18 years) accounted for 39.2% (n=196; 95% of whom were <5 years), adults (19–60 years) for 15.4% (n=77), and older adults (>60 years) for 45.4% (n=227). The majority of strains (51.8%) were sourced from the nine principal districts of cities, but a lesser fraction (48.2%) was derived from the counties. In the central urban districts, resistance rates to ampicillin, amoxicillin/clavulanate, azithromycin, and tetracycline were 76.8%, 59.4%, 42.1%, and 8.1%, respectively, which were higher than those in surrounding districts/counties (75.5%, 56.4%, 34.4%, and 4.1%); however, none of these differences reached statistical significance (*p >*0.05) (**Table 1**).

**Table 1:** Basic characteristics and antimicrobial resistance profiles of *H. influenzae* isolates in Chongqing, China.

Hospital	Cases	Sex		Population			Antibiotics R n(%)							
		Female	Male	Children	Adult	Elderly	AMP	AMC	CAZ	CTX	AZI^*^	LVF^*^	MER	TET
Second Affiliated Hospital of Chongqing Medical University	121	41	80	0	39	82	95(19.0)	27(5.4)	0	0	51(10.2)	1(0.2)	0	14(2.8)
Children’s Hospital of Chongqing Medical University	77	32	45	77	0	0	59(11.8)	25(5.0)	0	0	37(7.4)	0	0	5(1.0)
Southwest Hospital of Army Medical University	33	11	22	25	3	5	24(4.8)	9(1.8)	0	0	9(1.8)	2(0.4)	0	1(0.2)
Second Affiliated Hospital of Army Medical University	28	4	24	5	6	17	21(4.2)	6(1.2)	0	0	12(2.4)	0	0	1(0.2)
Jiangjin District Central Hospital	55	19	36	30	9	16	42(8.4)	12(2.4)	0	0	16(3.2)	0	0	3(0.6)
People’s Hospital of Dianjiang County	28	5	23	14	2	12	16(3.2)	5(1.0)	0	0	9(1.8)	0	0	0
People’s Hospital of Shizhu County	11	5	6	1	1	9	6(1.2)	2(0.4)	0	0	1(0.2)	0	0	0
People’s Hospital of Zhong County	11	6	5	3	1	7	8(1.6)	3(0.6)	0	0	4(0.8)	0	0	0
People’s Hospital of Fengdu County	136	51	85	41	16	79	110(22.0)	30(6.0)	0	0	52(10.4)	0	0	7(1.4)

*: non-susceptibility; AMP, Ampicillin; AMC, Amoxicillin/Clavulanate; CAZ, Ceftazidime; CTX, Cefotaxime; AZI, Azithromycin; LVF, Levofloxacin; MER, Meropenem; TET, Tetracycline; Children<18 years;Elderly >60years. Chi-square test was used for categorical variables; Fisher’s exact test was used when expected cell counts were <5 (or when assumptions for chi-square were not met).

### 3.2 Antimicrobial susceptibility

Among the 500 isolates included in this study, the resistance rates to ampicillin, azithromycin, amoxicillin-clavulanate, levofloxacin, and tetracycline were 76.2%, 38.2%, 23.8%, 0.6%, 6.2%, respectively. In contrast, all isolates were susceptible (100%) to third generation cephalosporins and the carbapenem tested, including ceftazidime, cefotaxime, and meropenem. Compared with isolates from adult patients, isolates from pediatric patients exhibited higher resistance rates to ampicillin, amoxicillin–clavulanate, and azithromycin (*p* = 0.100, 0.771, and 0.036, respectively); only the difference in azithromycin resistance reached statistical significance (*p* < 0.05) (**Table 2**).

**Table 2:** The susceptibility of H. influenzae to antibacterial drugs(%).

Antibiotics	Total strains(n=500)		MIC(mg/L)			Children(n=196)		Adults(n=304)		*p*
	R	S	Range	MIC50	MIC90	R	S	R	S	
Ampicillin	76.2	20.6	0.25-32	32	32	80.1	16.8	73.7	23.7	0.100
Amoxicillin/Clavulanate	23.8	72.4	1-32	2	32	24.5	71.9	23.4	72.7	0.771
Ceftazidime	0.0	100.0	0.125-2	0.5	1	0.0	100.0	0.0	100.0	—
Cefotaxime	0.0	100.0	0.125-1	0.25	1	0.0	100.0	0.0	100.0	—
Azithromycin	38.2*	61.8	0.25-32	1	32	43.9*	56.1	34.5*	65.5	0.036
Levofloxacin	0.6*	99.4	0.125-16	0.125	0.5	0.5*	99.5	0.7*	99.3	0.100
Meropenem	0.0	100.0	0.03-0.5	0.25	0.5	0.0	100.0	0.0	100.0	—
Tetracycline	6.2	91.6	0.25-16	0.5	1.0	6.1	88.8	6.3	93.4	0.954

*: non-susceptibility; children and adult: Chi-square test was used for categorical variables; Fisher’s exact test was used when expected cell counts were <5 (or when assumptions for chi-square were not met).

### 3.3 Minimum inhibitory concentration distribution characteristics

**Figure 1:**
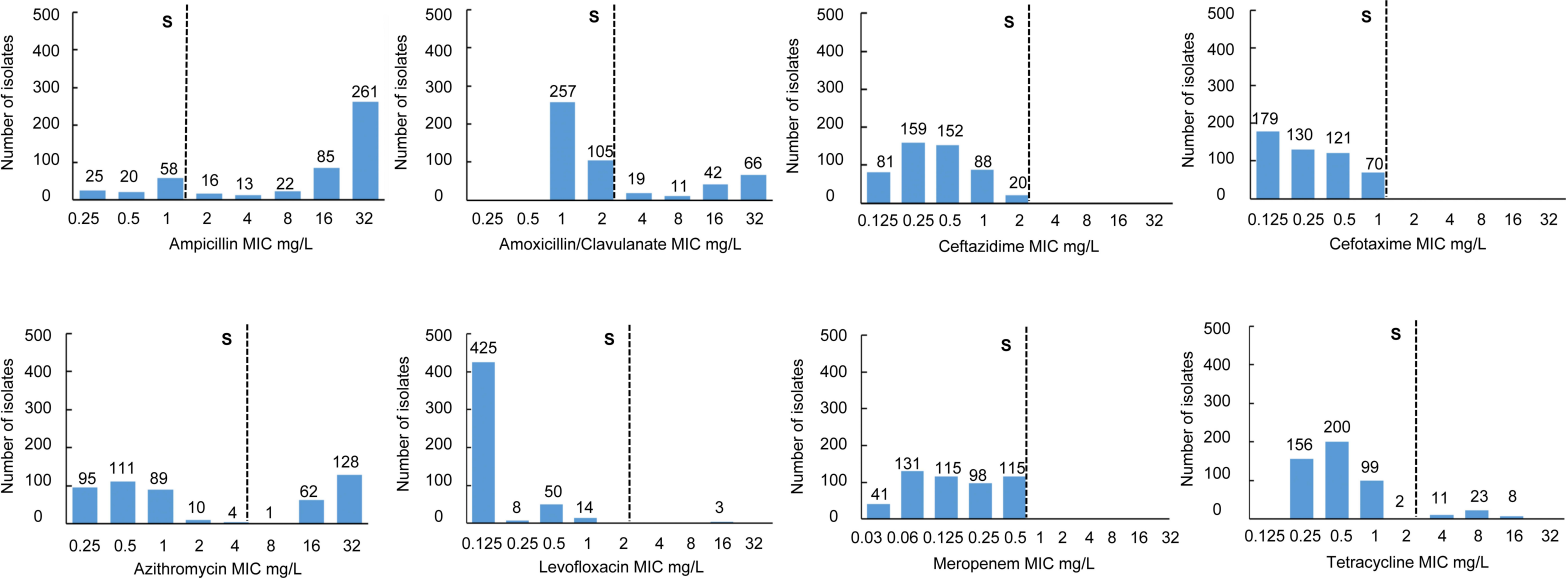
Correlation between antimicrobial concentration and *H. influenzae* strain counts: a bar chart analysis.

Among the 500 *H. influenzae* isolates analyzed, ampicillin MICs ranged from 0.25 to 32 mg/L and showed a high level of resistance; 346 isolates had MICs ≥16 mg/L. For amoxicillin–clavulanate, MICs were mainly distributed between 0.5 and 2 mg/L, but a high-MIC tail extending to 32 mg/L was observed. Azithromycin had a high-MIC peak at 32 mg/L (128 isolates); 191 isolates had MICs ≥8 mg/L. In contrast, levofloxacin, third-generation cephalosporins (ceftazidime/cefotaxime), and meropenem showed low MICs with a markedly left-skewed distribution, indicating high *in vitro* activity against the isolates. Tetracycline MICs were mostly 0.25–1 mg/L, while a high-MIC tail was present at 4–16 mg/L (**Figure 1**).

### 3.4 β-lactamase phenotype

Among the 500 isolates, 67.2% (336/500) were β-lactamase–positive, whereas 32.8% (164/500) were β-lactamase–negative. The resistance rates to ampicillin, amoxicillin–clavulanate, azithromycin, and tetracycline were significantly higher in β-lactamase–positive isolates than in β-lactamase–negative isolates (*p* < 0.001). Notably, no resistance to azithromycin or tetracycline was detected among β-lactamase–negative strains, corresponding to a susceptibility rate of 100% (**Table 3**).

**Table 3:** Comparison of antimicrobial resistance profiles between β-lactamase-producing and non-producing *H. influenzae* strains(%).

Antibiotics	β-lactamase positive, n=336		β-lactamase negative, n=164		*p*
	R	S	R	S	
Ampicillin	336(100)	0.0	45(27.4)	103(62.8)	<0.001
Amoxicillin/Clavulanate	105(31.3)	214(63.7)	14(8.5)	148(90.2)	<0.001
Ceftazidime	0.0	336(100.0)	0.0	164(100.0)	—
Cefotaxime	0.0	336(100.0)	0.0	164(100.0)	—
Azithromycin	191(56.8)*	145(43.2)	0.0	164(100.0)	<0.001
Levofloxacin	1(0.3)*	335(99.7)	2(1.2)*	162(98.8)	0.252
Meropenem	0.0	336(100.0)	0.0	164(100.0)	—
Tetracycline	31(9.2)	294(87.5)	0.0	164(100.0)	<0.001

*: Non-sensitivity rate;Chi-square test was used for categorical variables; Fisher’s exact test was used when expected cell counts were <5 (or when assumptions for chi-square were not met).

### 3.5 Analysis of antimicrobial resistance mechanisms

#### 3.5.1 Resistance mechanisms and genomic characteristics of H. influenzae isolates from Chongqing

**Figure 2:**
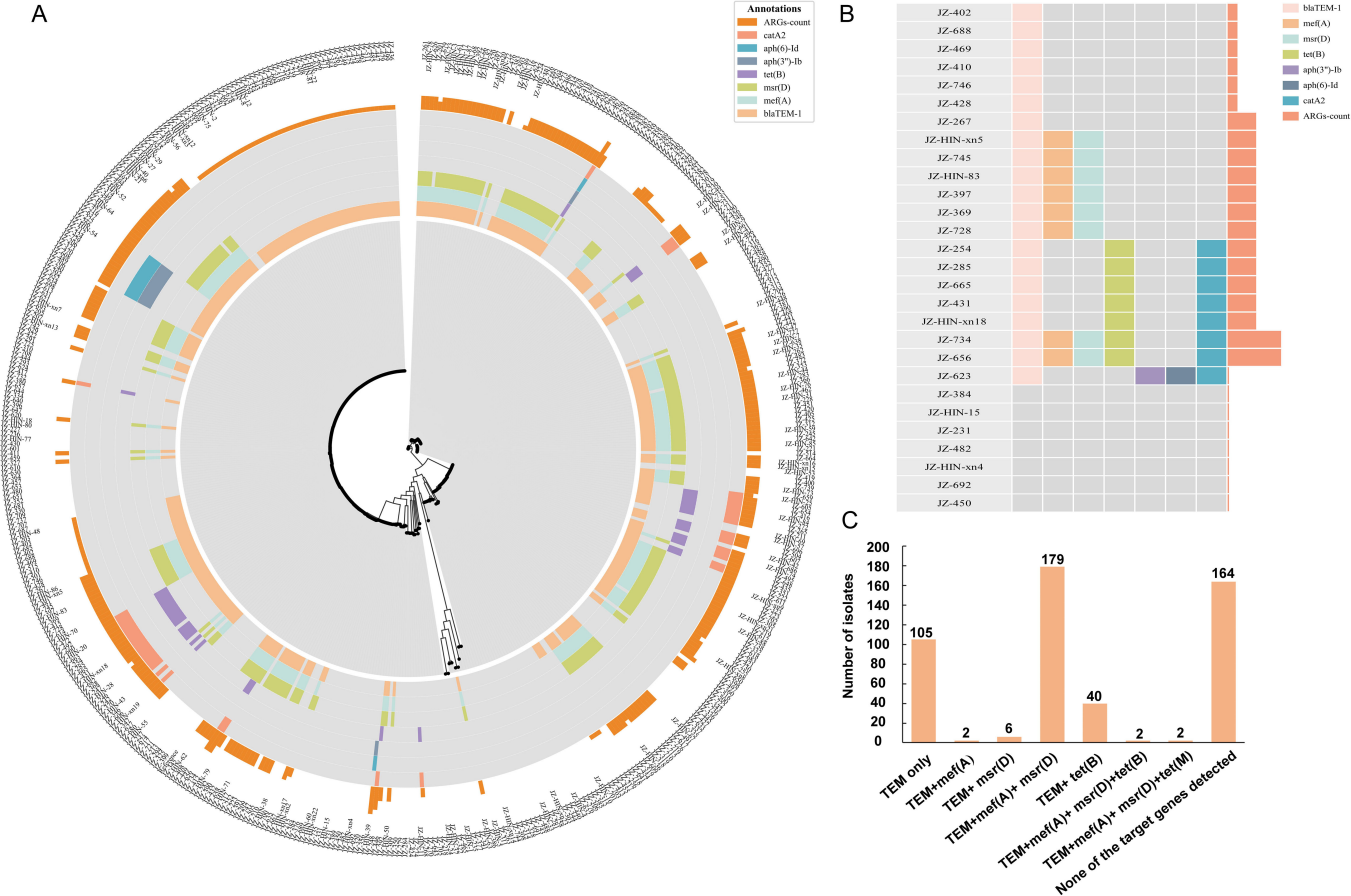
Circular phylogenetic tree of 500 clinical *H. influenzae* isolates from Chongqing. **(A)** Maximum-likelihood phylogeny inferred from core-genome SNPs. From the center outward, colored rings denote, in order: the core phylogeny, the specific ARG repertoire, and the ARG count; **(B)** Rectangular heatmap indicating presence/absence of representative antimicrobial resistance genes across selected isolates; **(C)** Summary of recurrent ARG combinations and the number of isolates carrying each combination.

Whole-genome assemblies showed that the 500 isolates had genome sizes of 1.8–2.0 Mb and a mean GC content of 38.0%. CheckM indicated >95% completeness for all genomes, with contamination <1.5%. The predicted number of coding sequences ranged from 1, 700 to 1, 900. A maximum-likelihood phylogeny inferred from core-SNP sites was visualized as a circular tree to depict overall relatedness among isolates. Screening of five target antimicrobial resistance genes (ARGs) showed that 164 isolates lacked all five genes, whereas 336 carried at least one. Notably, *blaTEM* was detected in 336 isolates, and the distribution of ARG-positive isolates displayed a clustering pattern on the phylogeny (**Figure 2A**). Co-occurrence of *blaTEM* with *mef(A)*/*msr(D)* and/or *tet(B)* was common (46.2%, 231/500), generating diverse ARG combinations (**Figure 2B**). These single- or multi-gene patterns formed the local core resistome (**Figure 2C**), providing a molecular basis that may facilitate sustained circulation of resistant *H. influenzae* in this region. Mobile genetic elements (MGEs) were frequently observed among ARG-positive isolates: 306 isolates (91.1%, 306/336) carried at least one MGE. The predominant insertion sequences were IS*Hpa1* (83.9%, 419/500), IS*Hps4* (2.2%, 11/500), IS*Aac3* (1.6%, 8/500), and IS*Spu10* (0.2%, 1/500) (**Figure 2**).

#### 3.5.2 Genomic characteristics of resistant H. influenzae strains in China

**Figure 3:**
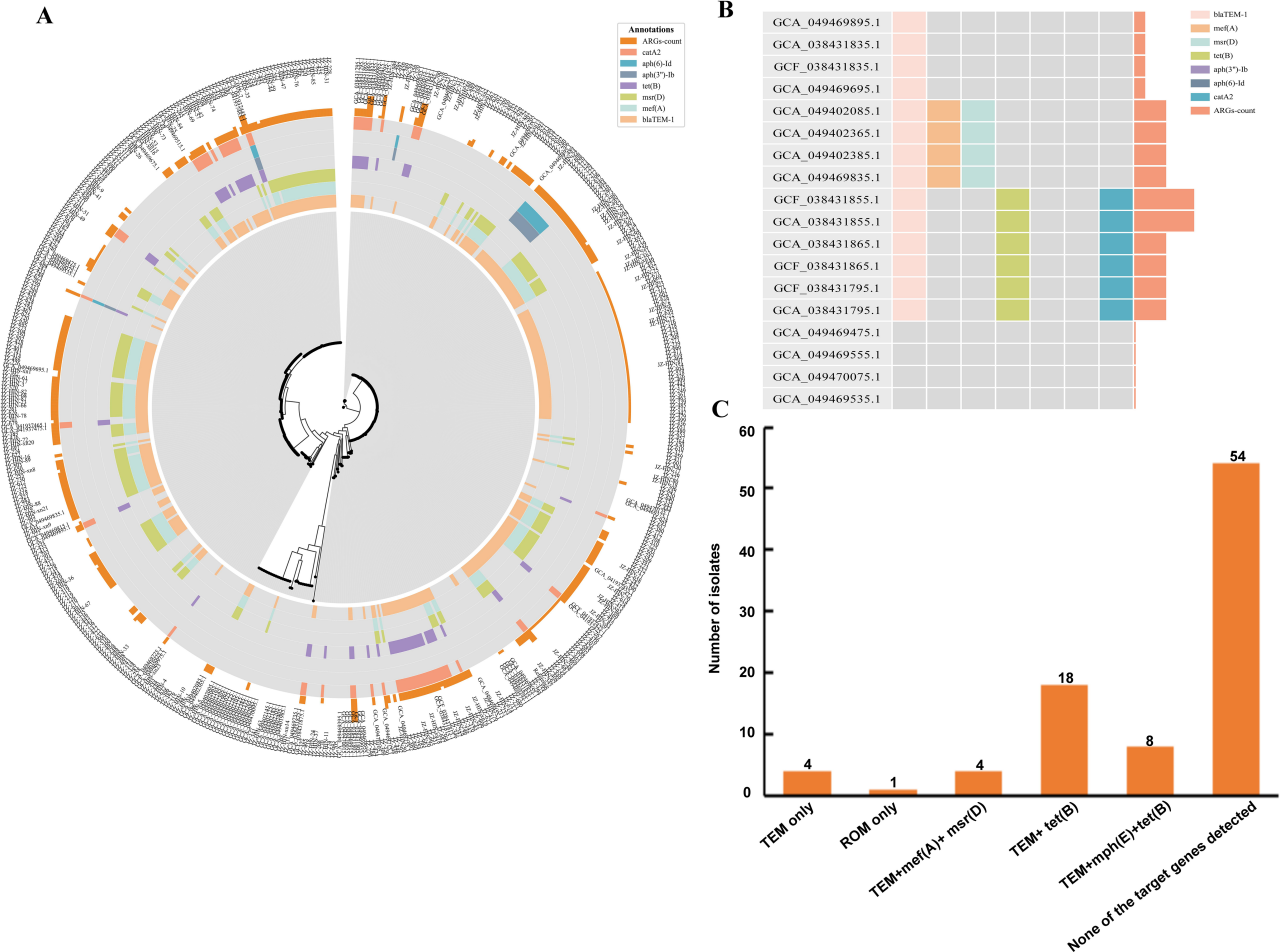
Circular phylogeny of *H. influenzae* from the national genomic dataset (n=89) and Chongqing (n=500); **(A)** Maximum-likelihood phylogeny inferred from core-genome SNPs. From the center outward, colored rings denote, in order: the core phylogeny, the specific ARG repertoire, and the ARG count; **(B)** Rectangular heatmap indicating presence/absence of representative antimicrobial resistance genes across selected isolates; **(C)** Summary of recurrent ARG combinations and the number of isolates carrying each combination.

**To evaluate the phylogenetic** status of local prevalent strains at the national level, this study performed a comparative genomic analysis of the chromosome genomes of 500 locally isolated strains and 89 representative *H. influenzae* strains of mainland Chinese origin retrieved from public databases. A maximum-likelihood phylogenetic tree derived from core-genome SNPs demonstrated a different population structure (**Figure 3A**). The Chongqing isolates were not confined to a single clade but rather dispersed across multiple phylogenetic clusters, intermingling with strains from other regions. Meanwhile, a relative aggregation of Chongqing-derived strains was observed within several clusters. These findings indicate that the local prevalent strains are deeply integrated into the national *H. influenzae* population structure while exhibiting phylogenies with certain geographical preferences. Annotations of ARGs and their copy numbers overlaid on the outer ring of the tree demonstrated that multidrug-resistant (MDR) strains carrying bla*TEM-1* along with other resistance genes such as *mef(A)*, *msr(D)*, or *tet(B)* were predominantly clustered in specific evolutionary branches. In contrast, strains lacking the target ARGs or carrying only a limited number of resistance genes showed a more dispersed distribution pattern. This structure, characterized by the coexistence of high-resistance clonal clusters and a diverse susceptible background, underscores the critical role of clonal expansion in the local prevalence of drug resistance. Among all the national strains analyzed, 35 isolates harbored at least one resistance gene. The distribution of resistance genes was as follows: β-lactams: *TEM*-type (n=34), *ROB*-type (n=1); macrolides: *mef(A)* (n=4), *msr(D)* (n=4), *msr(E)* (n=1), *mph(E)* (n=8); tetracyclines: *tet(B)* (n=26). The *TEM*-type gene was identified as the dominant resistance determinant, which frequently co-occurred with the tetracycline resistance gene *tet(B)*, forming various stable resistance gene combinations (**Figures 3B, C**). Furthermore, 88.57% (31/35) of the ARG-positive strains were found to carry mobile genetic elements (MGEs). The predominant MGEs included insertion sequences IS*Hpa1* (58.4%, 52/89), IS*Hps4* (22.4%, 20/89), IS*Aac3* (10.1%, 9/89), and transposon *Tn6023* (3.3%, 3/89).

#### 3.5.3 Global evolutionary framework and shared molecular features of *H. influenzae* resistance

**Figure 4:**
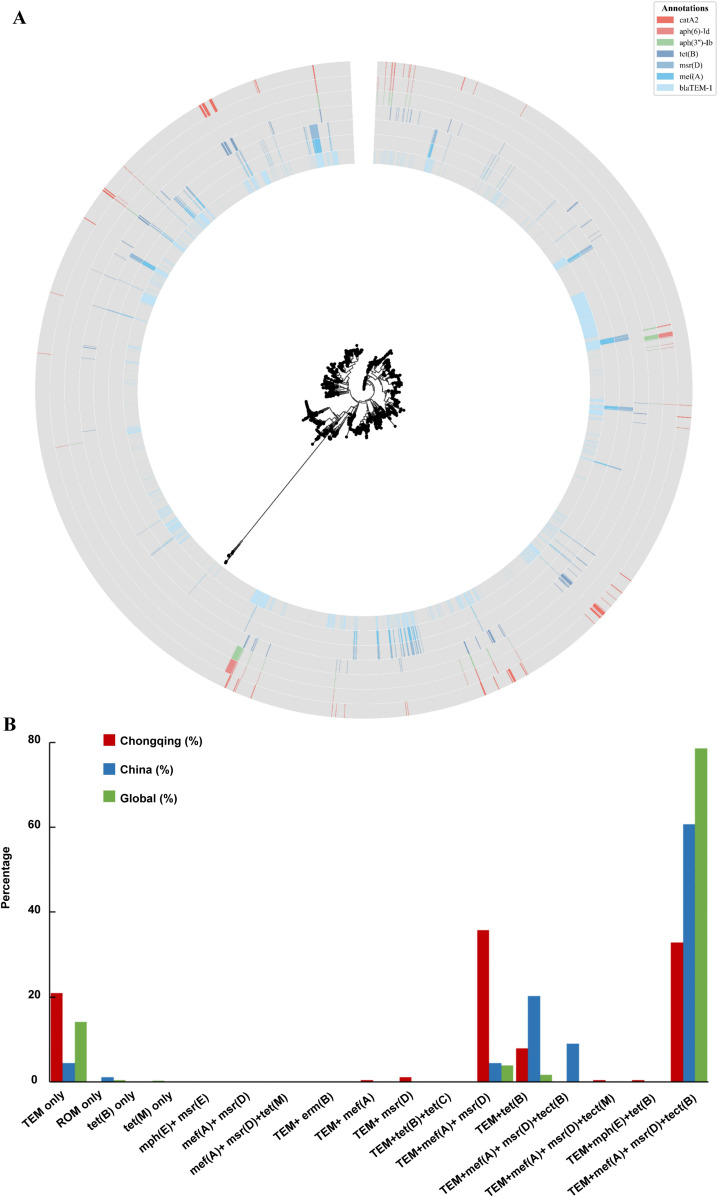
Circular phylogenetic tree and summary of antibiotic resistance genes (ARGs) for *H. influenzae* genomes from the global genomic database (n=5, 005), Chongqing (n=500), and China (n=89); **(A)** Maximum-likelihood phylogeny inferred from core-genome SNPs. From the center outward, colored rings denote, in order: the core phylogeny, the specific ARG repertoire, and the ARG count; **(B)** Rectangular heatmap indicating presence/absence of representative antimicrobial resistance genes across selected isolates; **(C)** Summary of recurrent ARG combinations and the number of isolates carrying each combination.

We further expanded the analysis to 5, 005 global isolates, among which 1, 070 carried at least one resistance gene. β-lactam resistance genes included *TEM*-type genes (n=1, 025) and *ROB*-type genes (n=25). Macrolide resistance genes included *mef(A)* (n=216), *msr(D)* (n=218), *msr(E)* (n=1), *mph(E)* (n=9), and *erm(B)* (n=1). Tetracycline resistance genes included *tet(B)* (n=117), *tet(M)* (n=12), and *tet(C)* (n=1). Phylogenetic mapping revealed lineage-associated clustering of resistance determinants (**Figure 4A**). *TEM* genes were the most prevalent and were frequently accompanied by macrolide resistance genes (*mef(A)*, *msr(D)*, *mph(E)*) and/or tetracycline resistance genes (*tet(B)*, *tet(M)*, *tet(C)*), yielding multiple composite ARG profiles (**Figure 4B**). Notably, 25 isolates carried *ROB* alone, 2 carried a macrolide resistance gene alone, 19 carried a tetracycline resistance gene alone, and 3 carried both macrolide and tetracycline resistance genes. Global data indicated that the majority of strains possessed mobile genetic elements (MGEs) (4268/5005, 85.3%), with strains harboring the *TEM* gene in conjunction with macrolides or the *TEM* gene alongside macrolides and tetracyclines accounting for MGEs at rates of (166/206, 80.6%) and (17/19, 89.5%) respectively. Meanwhile, a minority of resistant isolates lacked detectable MGEs (182/1, 070, 17%), which may reflect the genomic integration of resistance determinants via alternative mechanisms (e.g., natural recombination and DNA uptake) that no longer depend on intact MGE scaffolds, or limitations in current MGE detection approaches (**Figure 4**).

## 4 Discussion

*H. influenzae* is a Gram-negative bacterium that commonly colonizes the human upper respiratory tract as an opportunistic pathogen. Consistent with patterns reported in others (Wen et al., 2023; Tran et al., 2024), in our cohort the highest incidence occurred in children <5 years and adults ≥60 years; the oldest case was 92 years and the youngest was 33 days old. The elevated burden in older adults and young children may relate to impaired or immature immunity. The male-to-female ratio was 1.87:1, indicating higher susceptibility in males, a difference that may reflect the greater chronic obstructive pulmonary disease (COPD) burden among adult men in China (Wang et al., 2018) and the established relationship between NTHi airway colonization and COPD exacerbations (Short et al., 2021). As observed in many regions of China (Ma et al., 2023; Wang et al., 2025), cases in our area clustered in winter and spring, likely owing to abrupt cooling and damp conditions during the seasonal transition in Southwest China that heighten overall respiratory infection risk.

This study investigated the antibiotic susceptibility and MIC distribution of *H·influenzae* isolates from Chongqing. Our findings revealed significant variations in resistance rates to different β-lactam antibiotics. Specifically, ampicillin exhibited the highest resistance rate at 76.2%, which was consistent with the 2024 national average (CHINET, 2024). This rate was markedly higher than those reported in Japan (37.3% (Tokimatsu et al., 2025)) and several European countries, including Norway (18.6% (Tønnessen et al., 2022)), Belgium (18.9% (El Nouwar et al., 2025)), and Italy (21.7% (Giufrè et al., 2023)). MIC analysis further supports the above conclusion: The MIC range for ampicillin was 0.25–32 mg/L, with both the MIC_50_ and MIC_90_ reaching 32 mg/L, well above the susceptibility breakpoint. Further stratified analyses showed slightly higher ampicillin resistance in urban versus suburban districts (76.8% vs 75.5%), and markedly higher resistance in children than in adults (80.1% vs 73.7%, (*p* < 0.05)), paralleling national surveillance data (78.9% vs 69.1%). Based on previous surveillance data of bacterial resistance in Chongqing, the ampicillin resistance rate detected in this study was 76.2%, which was not only at a high level but also showed a continuous upward trend, significantly higher than 69.2% in 2020 and 73% in 2023 in Chongqing^2^. These findings highlight the alarmingly high and rapidly increasing ampicillin resistance in Chongqing, emphasizing the need for timely mitigation strategies. These findings collectively indicate that the situation of ampicillin resistance in Chongqing is extremely severe with a marked rising tendency, and it is imperative to formulate and implement targeted intervention and control strategies to curb the further spread of drug resistance. Resistance to amoxicillin–clavulanate was lower (23.8%) and broadly aligned with Japan (26.6% (Tokimatsu et al., 2025)), but higher than national, Norwegian (Tønnessen et al., 2022), and Italian (4.3% (Giufrè et al., 2023)) estimates, while remaining below that of France (47.4% (Taha et al., 2022)). Temporal and geographic variation likely contributes to differences in amoxicillin–clavulanate resistance. By contrast, resistance to third generation cephalosporins and to meropenem remains low in both national and global datasets (Tønnessen et al., 2022; Tokimatsu et al., 2025), and their MIC ranges were minimal.

Among the 500 *H. influenzae* isolates, only three were resistant to the fluoroquinolone levofloxacin (0.6%), a rate consistent with most domestic studies and with reports from Japan (0.0% (Tokimatsu et al., 2025)) and Sweden (2.4% (Aspevall et al., 2024)), indicating generally high fluoroquinolone susceptibility in Chongqing. For tetracyclines lacking national surveillance data the resistance rate was 6.2% in our cohort, broadly comparable to Sweden (0.6% (Aspevall et al., 2024)) and Norway (2.7% (Tønnessen et al., 2022)) but lower than the global average (19.9% (Abavisani et al., 2024)) and Ethiopia (42.86% (Kebede et al., 2022)). Azithromycin showed an MIC range of 0.25–32 mg/L with an MIC_90_ of 32 mg/L; the resistance rate was 38.2%, markedly lower than the national estimate (53.6% (CHINET, 2024)) yet much higher than Japan (0.0%) and the approximate global average (9.3%), while remaining below Vietnam (70.6% (Đỗ et al., 2025)). Within Chongqing, azithromycin resistance was higher in urban than county areas (42.1% vs 34.0%), and higher in children than adults (43.9% vs 34.5%, (*p* < 0.05)), patterns that may reflect differences in antimicrobial use, prevailing strain lineages, and local microbial ecology.

Genome-wide analysis of antimicrobial resistance mechanisms in *H. influenzae* isolates from Chongqing suggests that resistance determinants in this setting are largely linked to mobile genetic elements (MGEs), with many likely integrated into the chromosome. Comparative phylogenomic assessment against national and global collections further indicates that resistance gene carriage is non-random across the population structure, with resistant genotypes tending to aggregate within particular phylogenetic clusters rather than being evenly distributed. Within the Chongqing collection, *blaTEM-1* was the predominant β-lactamase gene and frequently co-occurred with macrolide-associated *mef(A)* and/or *msr(D)* and the tetracycline resistance gene *tet(B).* Notably, as shown in **Figure 2**, *mef(A)*, *msr(D)*, and *tet(B)* were detected exclusively among *blaTEM*-positive isolates, with no instances of these genes occurring independently in *blaTEM*-negative isolates. This apparent “*TEM*-dependent” resistance-gene combination pattern is consistent with the phenotypic susceptibility profiles in **Table 3**, where β-lactamase–negative isolates showed 0% resistance to both azithromycin and tetracycline. Together, these findings imply that, in the absence of β-lactamase, macrolide and tetracycline resistance remains uncommon in β-lactamase–negative isolates in this region, and/or that lineages carrying such determinants without *blaTEM* have not yet emerged as detectable population-level epidemic clusters. At the national scale, *blaTEM* also appears to remain the dominant β-lactamase determinant, most commonly observed in combination with *tet(B)*, whereas macrolide resistance genes such as *mef(A)* and *msr(D)* are detected at comparatively lower frequencies. In the global collection included in our analysis, *TEM*-type β-lactamases likewise predominate and frequently co-occur with macrolide resistance genes *(mef(A), msr(D))* and/or tetracycline resistance genes *(tet(B))*, while a small number of isolates carrying only macrolide- or tetracycline-associated genes are also present. This broader landscape raises the possibility that non–*TEM*-dependent resistance gene constellations may emerge and persist under different ecological or antimicrobial selection pressures, although further epidemiological evidence is required to clarify their transmission potential. Consistent with these observations, *ROB*-type β-lactamase genes were infrequently detected in both Chinese and global datasets. Regarding MGEs, most resistant isolates worldwide carried concurrent MGEs, whereas a minority lacked detectable MGEs. This may reflect either genomic integration of resistance determinants through mechanisms that no longer rely on intact, readily identifiable MGE scaffolds or methodological limitations in detecting structurally disrupted or atypical elements. Overall, the local core resistome in Chongqing is characterized by recurrent single- and multi-gene ARG combinations, providing a genomic framework for understanding the molecular basis of resistance in this region.

Whole-genome analysis indicated two principal mechanisms of ampicillin resistance in *H. influenzae*: β-lactamase production and mutations in *ftsI* leading to alterations of penicillin-binding protein 3 (PBP3). Among the 500 isolates, 336 were β-lactamase–positive (67.2%), comprising 336 carrying TEM; ROB was not detected, which was consistent with previous reports on the common β-lactamase profile of *H. influenzae* (Heinz, 2018).

Forty-five isolates were identified as β-lactamase-negative ampicillin-resistant (BLNAR) strains (9%) (**Table 3**). Overall, 11 distinct alleles were detected: *ftsI* allele 26 was the mostprevalent (n=21), followed by allele 146 (n=6). Amino acid sequence analysis of the transpeptidase domain of penicillin-binding protein 3 (PBP3) in these 45 isolates revealed multiple amino acid substitutions (**Supplementary Table 1**), consistent with previous reports on the PBP3 mutation spectrum associated with BLNAR (Sanbongi et al., 2006; García-Cobos et al., 2007). Most BLNAR isolates (28/45, 62.2%) were classified into the III-like+group, characterized by substitutions M377I, S385T, L389F, and R517H together with other substitutions. This was followed by the III+group (17/45, 37.8%), including 16 isolates with substitutions D350N, M377I, S385T, L389F, and N526K together with other substitutions, and one isolate with substitutions D350N, M377I, S385T, L389F, and N526H. BLNAR was first reported in Japan and has continued to rise annually in parts of Asia, Europe, and North America (García-Cobos et al., 2007; Skaare et al., 2014; Heinz, 2018). We also identified 105 β-lactamase positive ampicillin amoxicillin/clavulanate-resistant isolates (BLPACR; 21%), in which resistance is attributable to the coexistence of β-lactamase production and *ftsI* mutations (Sanbongi et al., 2006; García-Cobos et al., 2007).

The resistance of *H. influenzae* to fluoroquinolones is mainly derived fromalterations in the quinolone resistance-determining regions (QRDRs). In this study, after aligning the sequences with the Rd KW20 reference sequence (GenBank L42023.1) using SnapGene v6.0.2.0 software, it was found that all 3 levofloxacin-resistant *H. influenzae* strains had amino acid substitutions in the quinolone resistance-determining regions (QRDRs)(**Supplementary Table 2**). Among them, all strains with a levofloxacin MIC of 16 mg/L carried *GyrA* Ser84Leu, Asp88Tyr double mutations and *ParC* Ser84Ile substitution, suggesting that their high-level fluoroquinolone resistance is jointly mediated by key site mutations in *GyrA* combined with homologous site mutations in *ParC*, which is consistent with the previously clarified molecular resistance mechanism (Giufrè et al., 2023; Cherkaoui et al., 2025).

Whole-genome sequencing results further showed that the above 3 strains were all multidrug-resistant strains, which were collectively resistant to ampicillin, amoxicillin/clavulanic acid, and levofloxacin. Genetically, all strains carried *ftsI* mutations (such as Ser385Thr, Asn526Lys, etc.), *TEM*, as well as macrolide efflux-related genes *msr(D)* and *mef(A)*. This indicates that the multidrug-resistant phenotype is synergistically mediated by a variety of drug-resistant genotypes, and the combination of related drug-resistant genotypes has also been reported in previous studies (Cherkaoui et al., 2025).

We acknowledge that the associations between specific genotypes and resistance phenotypes identified in this study are based on genomic inference and require functional validation. While our observations are consistent with experimentally validated mechanisms reported in other studies — for example, *TEM-1*-mediated ampicillin hydrolysis in H. influenzae (Tristram et al., 2007), mef(A)-mediated macrolide efflux (Roberts et al., 1999), horizontal transfer of *fts*I between H. influenzae strains at frequencies of 10^-6^ to 10^-7^ (Takahata et al., 2007), and PBP3 substitution-driven beta-lactam resistance (Ubukata et al., 2001; Tristram et al., 2007) — the specific contributions of these determinants in our Chongqing isolates have not been experimentally confirmed. The co-occurrence of *blaTEM* with *mef(A)*/*msr(D)*/*tet(B)* as a putative core resistome module is a genomic observation that suggests, but does not prove, coordinated co-selection; experimental approaches such as conjugation assays, plasmid curing, and transcriptomic analysis would be needed to confirm functional linkage. Future studies should prioritize: (1) functional characterization of the *blaTEM*–*mef(A)*/*msr(D)*–*tet(B)* co-resistance module through conjugation experiments and fitness cost analyses (San Millan et al., 2010); (2) site-directed mutagenesis of novel *ftsI* alleles to confirm their contribution to beta-lactam resistance (Tristram et al., 2007); (3) experimental evolution studies under antibiotic selective pressure to identify evolutionary pathways to resistance (Petersen et al., 2025); and (4) transcriptomic profiling under antibiotic stress to elucidate regulatory mechanisms governing resistance gene expression.

Vaccination, serotype dynamics, and implications for resistance evolution. The introduction of the *H. influenzae* type b (Hib) conjugate vaccine has dramatically reduced invasive Hib disease worldwide (Watt et al., 2009). However, this success has been accompanied by a shifting epidemiological landscape, with non-typeable *H. influenzae* (NTHi) emerging as the predominant cause of respiratory tract infections, otitis media, and exacerbations of chronic obstructive pulmonary disease (Van Eldere et al., 2014). In our study, the vast majority of isolates were NTHi, consistent with the post-vaccine epidemiological transition observed globally. This shift has important implications for antimicrobial resistance, as NTHi strains lack the capsular antigens targeted by the Hib vaccine and are not subject to vaccine-mediated selective pressure on capsule loci. Instead, NTHi populations may face disproportionate antibiotic selective pressure due to increased recognition and treatment of NTHi infections, potentially accelerating resistance evolution. Our phylogenomic analysis revealed several concerning patterns relevant to resistance spread. At the local level, we identified clonal clusters of multidrug-resistant NTHi within and across hospitals in Chongqing, suggesting nosocomial or community transmission of resistant lineages. At the national scale, Chongqing isolates were phylogenetically interspersed with isolates from other Chinese provinces, indicating inter-regional dissemination of resistant clones. At the global scale, certain sequence types carrying the *blaTEM*–*mef(A)*/*msr(D)*–*tet(B)* resistance module were shared between Chinese and international isolates, raising the possibility of intercontinental resistance gene flow, likely mediated by mobile genetic elements such as ISHpa1. The current absence of a licensed NTHi vaccine poses a significant public health challenge. Several NTHi vaccine candidates targeting conserved outer membrane proteins (e.g., Protein D, OMP P6) are under development (Poolman et al., 2000; Roier et al., 2012), and some have shown promise in clinical trials (Prymula et al., 2006). However, the high genomic diversity of NTHi, as demonstrated by our multi-scale phylogenetic analysis, poses challenges for vaccine antigen selection and suggests that strain coverage must be carefully evaluated. Moreover, the potential for vaccine-driven antigenic variation — analogous to serotype replacement observed in Streptococcus pneumoniae following PCV introduction (Weinberger et al., 2011) — warrants monitoring as NTHi vaccine candidates advance through clinical development. From a resistance containment perspective, our findings underscore the need for integrated strategies combining antimicrobial stewardship with genomic surveillance of resistant clonal lineages.

Macrolide resistance is generally driven by ribosomal target modification (Tristram et al., 2007), yet all azithromycin-resistant isolates here harbored *msr(D)* and/or *mef(A)*, indicating efflux-mediated resistance as the dominant mechanism; no integrative elements or transposons were detected, suggesting limited mobility of these determinants. Tetracycline resistance has been associated with the *tet(B)* efflux pump, ribosomal protection proteins, and chromosomal changes reducing outer-membrane permeability (Tristram et al., 2007; Soge and Roberts, 2011); all 31 tetracycline-resistant isolates carried *tet(B)*, implicating it as the major local mechanism, whereas two tetracycline-susceptible isolates possessed *tet(M)* alone, consistent with genotype–phenotype discordance (Rebelo et al., 2022). The strains in this study were primarily obtained from lower respiratory tract specimens of hospitalized patients, excluding outpatient and healthy carrier populations, resulting in certain limitations regarding sample representativeness. Antimicrobial susceptibility results were interpreted according to CLSI criteria only, and differences in interpretive standards and methodological variations may affect phenotypic determination. In addition, incomplete serotyping and vaccination information limited further analysis of the epidemiological profiles, pathogenic mechanisms, and vaccine impact on the bacterial strains. Additionally, longitudinal sampling would be needed to directly observe the dynamics of resistance emergence and clonal replacement over time, and to assess whether vaccine introduction or changes in prescribing practices alter resistance trajectories in this population.

## 5 Conclusions

In Chongqing, the predominant resistances observed in *H. influenzae* were to ampicillin, azithromycin, and tetracycline. Ampicillin resistance is principally mediated by *TEM-1* β-lactamase and *ftsI* (PBP3) mutations, yielding BLNAR and BLPACR phenotypes. Azithromycin resistance is partly driven by acquisition of the efflux determinants *msr(D)* and *mef(A)*, whereas tetracycline resistance was primarily driven by *tet(B)*.

Notably, no macrolide or tetracycline resistance genes were detected among β-lactamase-negative isolates, highlighting the need for accurate and continuous β-lactamase surveillance to support resistance risk assessment and therapeutic decision-making. Based on the local resistance profile, empirical therapy should be adjusted accordingly, with preference given to regimens expected to cover resistant isolates, such as β-lactam/lactamase inhibitor combinations or third-generation cephalosporins. Rational antibiotic use and standardized antimicrobial stewardship are essential to curb further escalation of *H. influenzae* resistance.

## Data Availability

The original contributions presented in the study are publicly available. This data can be found here: https://www.ncbi.nlm.nih.gov/; PRJNA1466135.

## Footnotes

1 https://www.bioinformatics.babraham.ac.uk/projects/fastqc/, (Accessed January 12, 2026).

2 http://www.carss.cn/ (Accessed February 27, 2026).

## Supplementary material

Supplementary Table 1 Deduced amino acid substitutions in the transpeptidase domain of PBP3 protein from 45 BLNAR isolates, Chongqing, China, 2024–2025.

Supplementary Table 2 Main characteristics of levofloxacin-resistant *Haemophilus influenzae* isolates: MICs, and QRDR substitutions.
