# Supplementary material for: Antimicrobial resistance characteristics and associated molecular mechanisms of clinically isolated Haemophilus influenzae from the lower respiratory tract in Chongqing, China
Source: Front Cell Infect Microbiol. 2026 Jun 3;16:1785829. doi: 10.3389/fcimb.2026.1785829 (PMC13272400; doi:10.3389/fcimb.2026.1785829)
Supplement: Supplementary Table 1 — Deduced amino acid substitutions in the transpeptidase domain of PBP3 protein from 45 BLNAR isolates, Chongqing, China, 2024–2025. [file Table1.docx]

**Supplementary Table 1.** Deduced amino acid substitutions in the transpeptidase domain of PBP3 protein from 45 BLNAR isolates, Chongqing,China, 2024–2025

| **Isolate** | **β-lac** | **AMP** | ***fts***I **Allele** | **PBP3 Group** | **D350N** | **S357N** | **M377I** | **S385T** | **L389F** | **V461I** | **A502V** | **V511A** | **R517H** | **N526K** | **T532S** |
| --- | --- | --- | --- | --- | --- | --- | --- | --- | --- | --- | --- | --- | --- | --- | --- |
| Hi4 | - | 8 | 26 | Ⅲ-like+ | * | * | * | * | * |  |  |  | * |  | * |
| Hi16 | - | 16 | 26 | Ⅲ-like+ | * | * | * | * | * |  |  |  | * |  | * |
| Hi37 | - | 8 | 26 | Ⅲ-like+ | * | * | * | * | * |  |  |  | * |  | * |
| Hi49 | - | 4 | 26 | Ⅲ-like+ | * | * | * | * | * |  |  |  | * |  | * |
| Hi60 | - | 8 | 107 | Ⅲ-like+ | * | * | * | * | * |  |  |  | * |  | * |
| Hi218 | - | 32 | 40 | Ⅲ+ | * | * | * | * | * |  |  |  |  | * |  |
| Hi237 | - | 4 | 26 | Ⅲ-like+ | * | * | * | * | * |  |  |  | * |  | * |
| Hi276 | - | 8 | 81 | Ⅲ-like+ | * | * | * | * | * |  |  |  | * |  | * |
| Hi287 | - | 16 | 40 | Ⅲ+ | * | * | * | * | * |  |  |  |  | * |  |
| Hi288 | - | 32 | 40 | Ⅲ+ | * | * | * | * | * |  |  |  |  | * |  |
| Hi290 | - | 8 | 26 | Ⅲ-like+ | * | * | * | * | * |  |  |  | * |  | * |
| Hi292 | - | 16 | 67 | Ⅲ+ | * | * | * | * | * |  | * |  |  | * |  |
| **Hi293** | - | 4 | 449 | Ⅲ-like+ | * | * | * | * | * |  |  |  | * |  | * |
| Hi294 | - | 4 | 197 | Ⅲ+ | * |  | * | * | * | * |  | * |  | H |  |
| Hi308 | - | 8 | 67 | Ⅲ+ | * | * | * | * | * |  |  |  |  | * |  |
| Hi316 | - | 4 | 26 | Ⅲ-like+ | * | * | * | * | * |  |  |  | * |  | * |
| Hi331 | - | 8 | 26 | Ⅲ-like+ | * | * | * | * | * |  |  |  | * |  | * |
| Hi336 | - | 4 | 146 | Ⅲ+ | * | * | * | * | * | * |  | * |  | * |  |
| Hi343 | - | 8 | 146 | Ⅲ+ | * | * | * | * | * | * |  | * |  | * |  |
| Hi356 | - | 8 | 157 | Ⅲ+ | * | * | * | * | * |  |  |  |  | * |  |
| Hi427 | - | 16 | 146 | Ⅲ+ | * | * | * | * | * | * |  | * |  | * |  |
| Hi433 | - | 32 | 40 | Ⅲ+ | * | * | * | * | * |  |  |  |  | * |  |
| Hi445 | - | 16 | 146 | Ⅲ+ | * | * | * | * | * | * |  | * |  | * | * |
| Hi448 | - | 4 | 26 | Ⅲ-like+ | * | * | * | * | * | * |  | * | * |  | * |
| Hi477 | - | 8 | 26 | Ⅲ-like+ | * | * | * | * | * | * |  | * | * |  | * |
| Hi480 | - | 8 | 26 | Ⅲ-like+ | * | * | * | * | * | * |  | * | * |  | * |
| Hi484 | - | 8 | 26 | Ⅲ-like+ | * | * | * | * | * | * |  | * | * |  | * |
| Hi488 | - | 8 | 67 | Ⅲ+ | * | * | * | * | * | * |  | * |  | * |  |
| Hi514 | - | 32 | 370 | Ⅲ-like+ |  |  | * | * | * | * |  | * | * |  | * |
| Hi615 | - | 4 | 26 | Ⅲ-like+ | * | * | * | * | * |  |  |  | * |  | * |
| **Hi622** | - | 4 | 450 | Ⅲ+ | * | * | * | * | * |  |  |  |  | * |  |
| Hi629 | - | 8 | 26 | Ⅲ-like+ | * | * | * | * | * |  |  |  | * |  | * |
| Hi634 | - | 4 | 26 | Ⅲ-like+ | * | * | * | * | * | * |  | * | * |  |  |
| Hi655 | - | 8 | 26 | Ⅲ-like+ | * | * | * | * | * |  |  |  | * |  |  |
| Hi689 | - | 8 | 107 | Ⅲ-like+ | * | * | * | * | * |  |  |  | * |  | * |
| Hi691 | - | 16 | 26 | Ⅲ-like+ | * | * | * | * | * | * |  | * | * |  | * |
| Hi692 | - | 8 | 441 | Ⅲ-like+ | * | * | * | * | * |  |  |  | * |  |  |
| Hi693 | - | 8 | 26 | Ⅲ-like+ | * | * | * | * | * | * |  | * | * |  | * |
| Hi706 | - | 4 | 26 | Ⅲ-like+ | * | * | * | * | * |  |  |  | * |  | * |
| Hi722 | - | 16 | 146 | Ⅲ+ | * | * | * | * | * | * |  | * |  | * |  |
| Hi735 | - | 16 | 107 | Ⅲ-like+ | * | * | * | * | * |  |  |  | * |  | * |
| Hi736 | - | 16 | 146 | Ⅲ+ | * | * | * | * | * | * |  | * |  | * |  |
| Hi742 | - | 32 | 26 | Ⅲ-like+ | * | * | * | * | * |  |  |  | * |  | * |
| Hi743 | - | 16 | 26 | Ⅲ-like+ | * | * | * | * | * |  |  |  | * |  | * |
| Hixn4 | - | 16 | 67 | Ⅲ+ | * | * | * | * | * |  |  |  |  | * |  |
| Isolates marked in bold carry newly submitted *fts*I alleles;AMP, ampicillin;PBP3 groups are III+, III-like+.An asterisk * indicates the presence of a substitution in the amino acid position indicated; H, substitution N526H. | | | | | | | | | | | | | | | |
