# Supplementary material for: Antimicrobial resistance characteristics and associated molecular mechanisms of clinically isolated Haemophilus influenzae from the lower respiratory tract in Chongqing, China
Source: Front Cell Infect Microbiol. 2026 Jun 3;16:1785829. doi: 10.3389/fcimb.2026.1785829 (PMC13272400; doi:10.3389/fcimb.2026.1785829)
Supplement: Supplementary Table 2 — Main characteristics of levofloxacin-resistant Haemophilus influenzae isolates: MICs, and QRDR substitutions. [file Table2.docx]

**Supplementary Table 2** Main characteristics of levofloxacin-resistant *Haemophilus influenzae* isolates: MICs, and QRDR substitutions

| **Isolate** | **β-lac** |  | **MIC (mg/mL)** | | | | | | | |  | **gyrA** | |  | **parC** |
| --- | --- | --- | --- | --- | --- | --- | --- | --- | --- | --- | --- | --- | --- | --- | --- |
|  |  |  | **AMP** | **AMC** | **CAZ** | **CTX** | **AZI** | **LVF** | **MER** | **TET** |  | **Ser-84** | **Asp-88** |  | **Ser-84** |
| Hi664 | + |  | 32 | 16 | 1 | 0.5 | 32 | 16 | 0.5 | 1 |  | Leu | Tyr |  | Ile |
| Hixn15 | + |  | 16 | 16 | 0.5 | 0.25 | 32 | 16 | 0.25 | 0.25 |  | Leu | Tyr |  | Ile |
| Hixn16 | + |  | 16 | 16 | 0.5 | 0.25 | 32 | 16 | 0.25 | 0.25 |  | Leu | Tyr |  | Ile |
| AMP:Ampicillin; AMC:Amoxicillin/Clavulanate; CAZ:Ceftazidime; CTX:Cefotaxime; AZI:Azithromycin; LVF:Levofloxacin; MER:Meropenem; TET:Tetracycline. | | | | | | | | | | | | | | | |
