# Supplementary material for: Antimicrobial resistance characteristics and associated molecular mechanisms of clinically isolated Haemophilus influenzae from the lower respiratory tract in Chongqing, China
Source: Front Cell Infect Microbiol. 2026 Jun 3;16:1785829. doi: 10.3389/fcimb.2026.1785829 (PMC13272400; doi:10.3389/fcimb.2026.1785829)
Supplement: Supplementary file 3 [file Table3.docx]

**Supplementary Table 3.** Summary of bioinformatics tools, versions, and key parameters used in this study.

| **Analysis Step** | **Tool** | **Version** | **Key Parameters** | **Input** | **Output** |
| --- | --- | --- | --- | --- | --- |
| Read quality assessment | FastQC | v0.11.9 | Default | Raw FASTQ reads | Quality report (HTML) |
| Read trimming & filtering | fastp | v0.23.4 | --qualified_quality_phred 20 --length_required 50 --cut_front --cut_tail --thread 4 | Raw FASTQ reads | Filtered FASTQ reads; QC report (JSON/HTML) |
| De novo genome assembly | SPAdes | v3.15.5 | --careful --cov-cutoff auto -t 16 | Filtered FASTQ reads | Assembled contigs (FASTA) |
| Assembly quality assessment | QUAST | v5.2.0 | Default; --min-contig 500 | Assembled contigs | Assembly statistics (N50, L50, total length, etc.) |
| Contig filtering | Custom script | — | Remove contigs <1,000 bp or mean coverage <5× | Assembled contigs | Filtered contigs (FASTA) |
| Genome completeness & contamination | CheckM | v1.2.2 | lineage_wf; completeness >95%, contamination <5% | Filtered contigs | Completeness/contamination estimates |
| Taxonomic assignment | GTDB-Tk | v2.6.1 | classify_wf; GTDB r220 database | Filtered contigs | Species classification (H. influenzae confirmed) |
| Genome annotation | Prokka | v1.14.6 | --kingdom Bacteria --genus Haemophilus --species influenzae --rfam --cpus 8 | Filtered contigs | GFF3, GenBank, protein FASTA |
| Acquired AMR gene detection | ResFinder | v4.1 | Sequence identity >90%; coverage >80% | Assembled contigs | AMR gene hits (TSV); allele assignments |
| Chromosomal point mutation detection | PointFinder | v4.1 | Species: Haemophilus influenzae; default thresholds | Assembled contigs | Point mutation report (TSV) |
| ftsI allele extraction & classification | BLASTn + custom script | 2.14.0+ | Query: ftsI transpeptidase domain vs Rd KW20 (L42023.1); ≥95% coverage, ≥1% identity difference for allele discrimination | Annotated genomes | ftsI allele assignments; PBP3 amino acid substitutions |
| Mobile genetic element detection | MobileElementFinder | v1.0.3 | Default database (mge_db) | Assembled contigs | IS elements, transposons, ICE annotations |
| Read mapping & variant calling | Snippy | v4.6.0 | --mincov 10 --minfrac 0.9 --ref FDAARGOS_1560 (CP085952.1) --cpus 8 | Filtered FASTQ + reference genome | Per-isolate VCF; core alignment |
| Recombination filtering | Gubbins | v3.2.1 | --iterations 5 --filter-percentage 25 | Core genome alignment | Recombination-filtered alignment |
| Core SNP extraction | SNP-sites | v2.5.1 | -c (core sites only) | Recombination-filtered alignment | Core SNP alignment (FASTA) |
| ML phylogeny (≤589 genomes) | IQ-TREE 2 | v2.2.0 | -m GTR+F+I+G4 -bb 1000 -alrt 1000 -nt AUTO | Core SNP alignment | Newick tree; bootstrap support values |
| ML phylogeny (5,594 genomes) | FastTree | v2.1.11 | -gtr -gamma -fastest | Core SNP alignment (global) | Newick tree; SH-like support values |
| Tree visualization & annotation | iTOL | v6.0 | Circular layout; annotation layers: ARGs, MGEs, metadata | Newick tree + annotation files | Annotated phylogenetic figures (PDF/SVG) |
| Resistance gene heatmap | R / pheatmap | v1.0.12 | Clustering: complete linkage; distance: binary | Gene presence/absence matrix | Heatmap figure (PDF) |
| Statistical analysis | SPSS | v26.0 | Chi-square test / Fisher exact test; significance: p < 0.05 | Antimicrobial susceptibility data | Statistical test results; p-values |
| Sequence alignment (ftsI QRDR) | SnapGene | v6.0.2 | Pairwise alignment vs Rd KW20 (L42023.1) | ftsI / gyrA / parC sequences | Amino acid substitution annotations |

*Note: All tools were executed on a Linux server (CentOS 7, 512 threads, 1.1 TB RAM). Default parameters were used unless otherwise specified. Custom scripts for data processing and figure generation are available in the GitHub repository. AMR, antimicrobial resistance; ARG, antibiotic resistance gene; ICE, integrative and conjugative element; IS, insertion sequence; MGE, mobile genetic element; ML, maximum likelihood; QRDR, quinolone resistance-determining region; SNP, single nucleotide polymorphism.*
